# RGD_PLGA Nanoparticles with Docetaxel: A Route for Improving Drug Efficiency and Reducing Toxicity in Breast Cancer Treatment

**DOI:** 10.3390/cancers15010008

**Published:** 2022-12-20

**Authors:** Enza Di Gregorio, Chiara Romiti, Antonino Di Lorenzo, Federica Cavallo, Giuseppe Ferrauto, Laura Conti

**Affiliations:** Department of Molecular Biotechnology and Health Sciences, University of Turin, Via Nizza 52, 10126 Turin, Italy

**Keywords:** breast cancer, Docetaxel, PLGA nanoparticles, magnetic resonance imaging, RGD, theranostic

## Abstract

**Simple Summary:**

Breast cancer is the most prevalent cancer in women and the leading cause of cancer-associated deaths. Although several therapeutic approaches are available, systemic chemotherapy remains the primary choice, especially for the treatment of advanced breast cancers. Unfortunately, systemic chemotherapy causes numerous side effects and damage to distal organs and requires high doses of drugs to achieve a therapeutic concentration in the tumor region. The use of nanosystems for drug delivery is a promising strategy to overcome these drawbacks. In this study, we developed Poly (lactic-co-glycolic acid) nanoparticles (PLGA-NPs) containing the chemotherapeutic drug Docetaxel, functionalized with a cyclic RGD tripeptide to allow the active targeting of α_v_β_3_ integrins, which are overexpressed in breast cancer. We demonstrated that PLGAs effectively deliver the drug to breast cancer cells in preclinical models, and are more effective than free Docetaxel in hampering tumor progression, while reducing side effects.

**Abstract:**

Breast cancer is the leading cause of cancer-related death in women. Although many therapeutic approaches are available, systemic chemotherapy remains the primary choice, especially for triple-negative and advanced breast cancers. Unfortunately, systemic chemotherapy causes serious side effects and requires high doses to achieve an effective concentration in the tumor. Thus, the use of nanosystems for drug delivery may overcome these limitations. Herein, we formulated Poly (lactic-co-glycolic acid) nanoparticles (PLGA-NPs) containing Docetaxel, a fluorescent probe, and a magnetic resonance imaging (MRI) probe. The cyclic RGD tripeptide was linked to the PLGA surface to actively target α_v_β_3_ integrins, which are overexpressed in breast cancer. PLGA-NPs were characterized using dynamic light scattering, fast field-cycling ^1^H-relaxometry, and ^1^H-nuclear magnetic resonance. Their therapeutic effects were assessed both in vitro in triple-negative and HER2+ breast cancer cells, and in vivo in murine models. In vivo MRI and inductively coupled plasma mass spectrometry of excised tumors revealed a stronger accumulation of PLGA-NPs in the RGD_PLGA group. Targeted PLGAs have improved therapeutic efficacy and strongly reduced cardiac side effects compared to free Docetaxel. In conclusion, RGD-PLGA is a promising system for breast cancer treatment, with positive outcome in terms of therapeutic efficiency and reduction in side effects.

## 1. Introduction

Breast cancer (BC) is the most common cancer in women, and its incidence is increasingly high [1]. Although noteworthy advances in diagnosis and therapy have improved patient prognosis in recent years, BC is still the primary cause of cancer-associated death in women in many countries [1]. Despite therapy, many patients progress to metastatic BC, a disease that is very difficult to treat [2].

Surgery, chemotherapy, radiotherapy, and hormone therapy represent the pillars of BC treatment, and several targeted therapies and immunotherapies are entering clinical practice [3]. Systemic chemotherapy based on anthracyclines or cyclophosphamide remains the main treatment for triple-negative (TN) BC, advanced BC, and high-risk luminal cancers [2]. Moreover, doxorubicin-based neoadjuvant chemotherapy is frequently used for operable tumors [4].

The onset of side effects and damage to distal organs are important issues related to the use of systemic chemotherapy [5]. The most common side effects, generally shared by all chemotherapeutic drugs, include gastrointestinal and skin disorders, neuropathies, nephropathies, suppression of bone marrow, fatigue, and hair loss [6]. Additionally, some other drug-specific side effects can occur [7]. Indeed, many chemotherapeutic drugs, including anthracyclines, taxanes, alkylating agents, antimicrotubule agents, and antimetabolites, are known to strongly affect cardiac output (CO) and ejection fraction (EF%), with severe consequences on patient health and life expectancy [8,9,10].

Therefore, important goals in cancer treatment are to reduce the dose of administered drugs, enhance their specific accumulation in the tumor site, and reduce the overall side effects. A challenge is presented by the transition from traditional to targeted chemotherapy, where the drug is specifically delivered at high doses to cancer cells [11,12].

Thus, approaches using biocompatible nanoparticles (NPs) targeting epitopes specifically overexpressed by tumor cells can be the best choice. Several drug-filled nanotherapeutic systems have already been tested at the preclinical level, and some have already entered clinical stages with hope for success [13,14,15,16]. Nanomedicine for cancer treatment has shown strong benefits in terms of efficacy, reduction in side effects and drug resistance, and good pharmacokinetics and biocompatibility.

Different classes of natural and/or synthetic chemicals can be employed to generate self-assembling and highly stable NPs, such as phospholipids (e.g., liposomes and micelles), lipids (e.g., nucleolipids), polysaccharides (e.g., cyclodextrins), nucleic acids, polypeptides (e.g., peptide-based nanoparticles) and polymers (e.g., PLGA and polymersomes) [17,18,19,20,21].

Among the available polymers, Poly (lactic-co-glycolic acid) (PLGA) is one of the most frequently used biodegradable and biocompatible synthetic polymers capable of generating very stable nanometric and spherical nanoparticles (PLGA-NPs) [22]. It is a Poly (lactic acid) (PLA) and Poly (glycolic acid) (PGA) block copolymer that is commercially available with different copolymer compositions (generally 50:50 PLA-PGA) and molecular weights. Owing to its biological and chemical properties (low toxicity, controlled and persistent release, and biocompatibility with cells and tissues), it has been approved by the US Food and Drug Administration and European Medicines Agency for human use in drug delivery systems [23]. The hydrolysis of PLGA leads to glycolic and lactic acids, which are non-immunogenic endogenous molecules that are easily metabolized by enzymes [22]. As generally adopted for all nanoparticles, including PLGA-NPs, the loading of polyethyleneglycol (PEG) moiety can be efficient in improving the pharmacokinetic properties of nanoparticles. In particular, PEG can: (i) improve NPs’ suspensibility and reduce aggregation, (ii) reduce uptake by macrophages, thus enhancing the blood half-life, and (iii) prevent nonspecific interactions with cell surfaces [24,25]. Indeed, it is well known that PEG chains create a hydrophilic protective layer around NPs, which reduces serum-dependent phagocytosis, by inhibiting the adsorption of opsonin proteins such as IgG and complement factors on NP surfaces [24,25].

PLGA-NPs can be very efficient in entrapping hydrophobic anticancer drugs, among which are molecules belonging to the taxane family (e.g., Docetaxel and Paclitaxel) or anthracycline family (e.g., Doxorubicin) that normally display low blood solubility [26,27,28,29].

Taxanes are considered one of the conventional treatments for BC and can significantly ameliorate patients’ progression-free and overall survival [26,27]. Their mechanism of action implies that they bind to tubulin and stabilize its polymerization. This blocks cell division, leading to cell cycle arrest and apoptosis. Despite their good therapeutic efficiency (either as single agentsor when administered in combination with other drugs), their pharmaceutical formulation remains a challenge. Taxanes contain lipophilic substituents that cause very poor aqueous solubility and stability [26,27]. The administration of taxane-containing NPs can overcome this drawback. Docetaxel (Taxotere^®^; Rhone-Poulenc Rorer, Inc., Paris, France) is one of the most commonly used drugs. It is a semisynthetic analog of Paclitaxel, originally synthesized starting from Baccatin III, a precursor molecule extracted from the needles of the European yew tree *Taxus baccata* [30]. The presence of two minor chemical structural modifications with respect to Paclitaxel makes it more favorable for preclinical and clinical applications [26,27]. Docetaxel has already been encapsulated inside PLGA-NPs with a good encapsulation rate [28,31].

NPs can passively deliver the drug to tumor cells through an enhanced permeability and retention (EPR) mechanism, which is typical of nanosized systems and leaky tumor endothelium [32].

However, in view of further enhancing proper delivery to the tumor region and limiting side effects to distal organs, active NPs targeting can be exploited [33,34,35].

The surface of PLGA-NPs can be chemically modified to host a targeting molecule capable of recognizing epitopes expressed on the tumor. A widely exploited possibility relies on the use of the cyclic Arg-Gly-Asp tripeptide (RGD), which targets integrin α_v_β_3_, commonly upregulated in inflammatory diseases and many solid cancers such as BC [36,37]. Integrin α_v_β_3_ interacts with the RGD sequence present in several extracellular matrix proteins and increases the invasiveness and metastasis of many solid cancers. Integrin α_v_β_3_ is considered a marker of tumor development, metastasis and neo-angiogenesis, because it is expressed at low levels in normal blood vessels and is overexpressed in tumor vessels and in many cancer cells [38]. The possibility of targeting integrin α_v_β_3_ using synthetic cyclic RGD makes this molecule an interesting target for tumor delivery systems.

Starting from the above-reported considerations, herein we exploited the feasibility of using RGD-PLGA-NPs filled with Docetaxel in BC models.

RGD-PLGA-NPs were prepared and extensively characterized from a chemo-physical point of view; then, their potentiality as a chemotherapeutic system was tested in vitro on murine BC cell lines. To this aim, cell lines belonging to two different tumor subtypes, the HER2^+^ TUBO cells [39] and the TN 4T1 cells, were used. Finally, orthotopic 4T1 TNBC murine models were generated and treated with Docetaxel-containing RGD-PLGA-NPs. The delivery of RGD-PLGA-NPs was followed by magnetic resonance imaging (MRI), upon encapsulation of an amphipathic Gadolinium (Gd)-based MRI contrast agent in the PLGA-NPs.

RGD-PLGA-NPs were compared with untargeted PLGA-NPs to check the effect of the targeting moiety on the in vitro and in vivo biodistribution of the NPs and on their efficiency.

## 2. Materials and Methods

### 2.1. Chemicals

**Figure 1:**
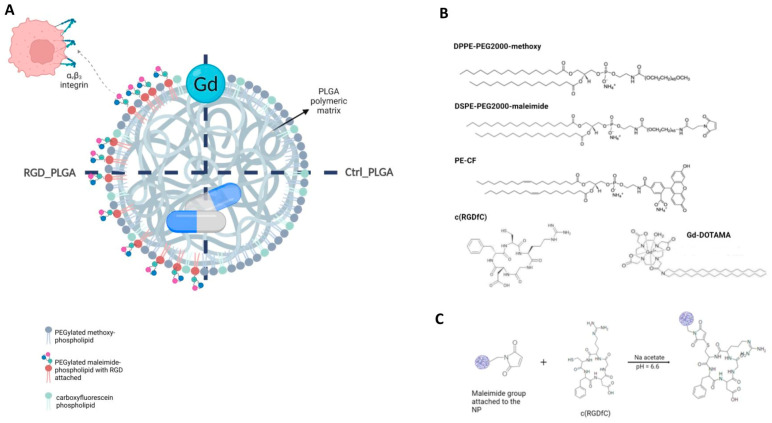
Generation of PLGA-NPs. (**A**) Structures of RGD_PLGA and Ctrl_PLGA-NPs. (**B**) Chemicals used for PLGA preparation. (**C**) Chemical reaction for c(RGDfC) ligation.

1,2-distearoyl-sn-glycero-3-phosphoethanolamine-N-[maleimide(polyethylene glycol)-2000] (ammonium salt) (DSPE-PEG2000-maleimide), 1,2-distearoyl-sn-glycero-3-phosphoethanolamine-N-[methoxy(polyethylene glycol)-2000] (ammonium salt) (DPPE-PEG2000-methoxy), and 1,2-dioleoyl-sn-glycero-3-pshosphoethanolamine-N-(carboxyfluorescein) (ammonium salt) (DOPE-CF) were purchased from Avanti Polar Lipids Inc. (Birmingham, AL, USA) (Figure 1B).

Poly(D,L-lactide-co-glycolide) (50:50, mol wt 30,000–60,000) (PLGA), poly vinyl alcohol (mol wt 31,000–50,000) (PVA), Trimethylsilylpropanoic acid (TSP, NMR standard) sodium chloride, sodium acetate, HEPES (4-(2-hydroxyethyl)-1-piperazineethane-sulfonic acid), sodium hydroxide, hydrochloric acid, nitric acid, chloroform, methanol, 3-(4,5-Dimethylthiazol-2-yl)-2,5-Diphenyltetrazolium Bromide, and all the other chemicals were acquired from Sigma-Aldrich Co. LLC (Burlington, NJ, USA) and used without further purification.

Cyclo(-Arg-Gly-Asp-D-Phe-Cys) acetate salt (c(RGDfC) was purchased from Bachem AG (Bubendorf, Switzerland).

Gd-amphiphilic complex, Gd(III)-DOTAMA (C18)2 (Gd-DOTAMA) was synthesized as previously reported [40]. Gd-ions were checked to be lower than 0.3% mol/mol by applying the orange xylenol procedure [41]. The exact concentration of Gd-complex was measured using a relaxometric approach, as previously reported [42]. Docetaxel (DTX) was purchased from Tocris Bioscience (Bristol, UK).

### 2.2. PLGA-NP Preparation

The method of the oil/water emulsion solvent extraction was used to prepare PLGA-NPs.

Briefly, the organic phase was prepared by dissolving Poly(D,L-lactide-co-glycolide) (50:50, mol/mol; *MW* = 30,000–60,000) (PLGA), pegylated phospholipid (DPPE-PEG(2000)methoxy) and all the other components in a chloroform/methanol 2:1 *v/v* mixture. For this purpose, 25 mg PLGA and 2.2 mg DPPE-PEG2000 methoxy—or, alternatively, for RGD-functionalized NPs, 1.0 mg DPPE -PEG2000 methoxy and 1.2 mg DSPE-PEG2000-Maleimide—were dissolved in 450 µL chloroform (purity ≥ 99.9%). 6.0 mg DTX (Docetaxel, drug) or 3.2 mg Gd-DOTAMA (MRI probe) were also added for Docetaxel- and Gd-loaded NP preparations, respectively [43,44]. Then, 50 µL of a solution containing DPPE-N-carboxyfluorescein (fluorescent probe) 1 mg/mL in pure chloroform was added for fluorescence microscopy exploitation.

The water phase was a poly vinyl alcohol (PVA) aqueous solution. PVA was chosen because it is a good emulsifier, able to produce NPs with a relatively uniform and small size, and is easily suspensible in water buffers [43,44]. The organic phase (0.5 mL) was gently dropped into 3 mL of aqueous phase containing 3% Poly (vinyl alcohol) (PVA), in ice. Sonication was performed with an immersion-tip instrument (SONOPULS HD 2070.2 Bandelin ultrasonic homogenizer, 20 KHz; Bandelin Electronic, Berlin, Germany) in 2 cycles of 1.5 min each, power 80–90%.

Evaporation of the organic solvent from the oil/water emulsion was carried out for 2 h under vacuum-rotation, and NPs were purified with centrifugation through filters and dialysis against fresh buffer [43,44].

Polymers and Gd-DOTAMA residuals were removed through dialysis at 6 °C against HEPES/NaCl buffer (1 L), under mild stirring (membrane with a 14 kDa MW cutoff). Centrifugation at 4 °C, 5500 rpm in Vivaspin^®^ tubes (300K MWCO) was used to wash the remaining PVA and concentrate the PLGA to the wanted volume (ca.3.5 mL). This was performed in HEPES/NaCl for Ctrl_RGD NPs and in Na^+^/acetate buffer 0.15 M (pH 6.6) for RGD NPs (diluting to 20 mL). Ctrl_RGD NPs were stored at 4 °C. pH of NPs for RGD ligation was checked after the concentration step, upon a 3-point calibration of the pHmeter (FiveEasyTM by Mettler Toledo), and pH was assessed to be around 6.6.

For RGD_PLGA, maleimide-pegylated phospholipid (DPPE-PEG (2000) maleimide) was added to the formulation and used to conjugate the cyclic RGD tripeptide (hereinafter indicated as c(RGDfC)) to the external surface of NPs, with the previously reported procedure [33] (Figure 1B, chemicals used for PLGA-NP preparation; Figure 1C, scheme of RGD binding). Ligation with c(RGDfC) was carried out by adding c(RGDfC) to the PLGA emulsion (5 folds stoichiometric excess of c(RGDfC) with respect to externally exposed maleimide moieties, in Na+/acetate buffer, pH 6.6) in a three-neck balloon, for 2 h, RT, under nitrogen flow and stirring [36]. Then, NPs were purified through dialysis (4 h in HEPES/NaCl). RG-functionalized NPs were also stored at 4 °C.

### 2.3. PLGA-NP Characterization, Quantification of Docetaxel Encapsulation and Release by NMR

The size of the formed nanoparticles was checked by using dynamic light scattering (DLS) with a Malvern Zetasizer Nano Series DLS (Malvern Panalytical, Malvern, UK) instrument, upon diluting NPs 1:100 in HEPES/NaCl buffer.

The amount of Docetaxel encapsulated in PLGA-NPs was measured by using high-resolution ^1^H-NMR, using a Bruker Avance 600 MHz (B0 = 14.09 T) spectrometer equipped with 5 mm probes and using CDCl3 as an internal lock spectrometer (Bruker, Billerica, USA). The temperature was controlled with Bruker thermostating units and fixed at 298 K. The experimental settings were the following: spectral width 30 ppm, 1024 scans, 4 dummy scans, water signal suppression, automatic RG, and recycle delay.

Preliminarily, a Docetaxel calibration curve was obtained by acquiring NMR spectra of Docetaxel at variable concentration (1.03–9.1 mg/mL) in CDCl3/MetOD (2:1 vol/vol) in the presence of 0.33 mg/mL of TSP (Trimethylsilylpropanoic acid) as a quantitative standard (Δδ of TSP = 0).

Three ^1^H signals of Docetaxel were considered for the preparation of the calibration curve and following calculation of Docetaxel in PLGA specimens, i.e., 5.68, 6.19, and 8.1 ppm (see Figures S2 and S3).

For analysis of PLGA and quantification of Docetaxel, 50 mL of Ctrl_PLGA or RGD_PLGA or Ctrl_PLGA were placed in glass tube and exsiccated under vacuum (using a rotavapor). Then, they were resuspended in 0.5 mL of CDCl3/MetOD (2:1 vol/vol) containing 0.33 mg/mL of TSP (Trimethylsilylpropanoic acid) as quantitative standard. ^1^H-NMR spectra of specimens were acquired as reported above and the intensity of peaks at 5.68, 6.19, and 8.1 ppm were measured, taking as reference the intensity of TSP (Δδ of TSP = 0).

Based on the calibration curve, the amount of encapsulated Docetaxel was calculated.

The encapsulation efficiency (EE%) of Docetaxel was calculated as follows:

(1) $${\mathrm{EE}}_{\mathrm{Docetaxel}}\%=\frac{{\mathrm{Docetaxel}}_{\mathrm{encap}}}{{\mathrm{Docetaxel}}_{\mathrm{tot}}}\times 100$$

where Docetaxel_encap_ was the amount of encapsulated Docetaxel (quantified as reported above) and Docetaxel_tot_ was the amount used for PLGA preparation.

### 2.4. Quantification of Gd-DOTAMA in PLGA-NPs and Assessment of PLGA-NPs’ Stability by 1H-Relaxometry

The encapsulation efficiency (EE%) of Gd-DOTAMA was calculated as follows:

(2) $${\mathrm{EE}}_{\mathrm{Gd}-\mathrm{DOTAMA}}\%=\frac{\mathrm{Gd}-{\mathrm{DOTAMA}}_{\mathrm{encap}}}{\mathrm{Gd}-{\mathrm{DOTAMA}}_{\mathrm{tot}}}\times 100$$

where Gd-DOTAMA_encap_ was the amount of encapsulated Gd-DOTAMA and Gd-DOTAMA_tot_ was the amount used for PLGA preparation.

The amount of encapsulated Gd-DOTAMA was calculated by quantifying the concentration of Gd through ^1^H-relaxometry. Briefly, Ctrl_PLGA or RGD_PLGA were mixed in equal volumes with 37% HCl and incubated overnight at 120 °C in sealed vials to solubilize the free Gd^3+^ aqua ion. The *R*_1_ values were measured using a Stelar SpinMaster relaxometer (Stelar, Mede, Italy) operating at 21.5 MHz. The inversion recovery method was used (16 experiments, two scans, recovery time ≥ 5 × T1). The reproducibility of the *R*_1_ data was ± 0.1%. Temperature (T = 25 °C) was regulated by a Stelar VTC-91 airflow heater, and the temperature inside the probe was measured using a calibrated digital thermometer (RS PRO RS55-11 RS, Sesto San Giovanni, Italy).

The concentration of Gd was determined using the following equation:

(3) $$R_{1}=R_{1d}+r_{1p}\times [{\mathrm{Gd}}^{3+}]$$

where *R*_1d_ is the diamagnetic contribution (0.5 s^−1^) and *r*_1p_ is the relaxivity of the free Gd^3+^ aqua ion (13.7 mM^−1^ s^−1^) under the same experimental conditions.

The stability of PLGA-NPs was assessed by monitoring over time the water proton longitudinal relaxation rates (*R*_1_) of Ctrl_PLGA or RGD_PLGA placed in HEPES/NaCl buffer or in human serum, at T = 37 °C.

### 2.5. ^1^H-Nuclear Magnetic Resonance Dispersion (NMRD) Profiles

Ctrl_PLGA and RGD_PLGA water proton longitudinal relaxation rates (*R*_1_) were measured as a function of the magnetic field strength in aqueous solutions using a fast field-cycling Stelar SmarTracer relaxometer (Stelar s.r.l., Mede, Italy), over a continuum of magnetic field strengths from 0.00024 to 0.25 T (0.01−10 MHz proton Larmor frequencies). This relaxometer works under computer control with an absolute uncertainty in 1/T_1_ of 1%. Additional longitudinal relaxation data in the range of 20−70 MHz were obtained using a Stelar relaxometer connected to a Bruker WP80 NMR electromagnet. The ^1^H T_1_ relaxation times were acquired using the standard inversion recovery method (with a 90° pulse width of 3.5 μs, 16 experiments, and 4 scans). The temperature was controlled by a Stelar VTC-91 airflow heater (Stelar, Mede, Italy) equipped with a calibrated copper−constantan thermocouple (uncertainty of ± 0.1 °C).

Data were reported as normalized millimolar relaxivity (*r*_1p_):

(4) $$r_{1p}=\frac{R_{1}-R_{1d}}{\left[{\mathrm{Gd}}^{3+}\right]}$$

### 2.6. Cell Cultures

4T1 cells were obtained in 2018 from the American Type Culture Collection (ATCC), stocked as frozen aliquots, then thawed and used for maximum 10 passages in RPMI 1640 (ThermoFisher Scientific Inc., Waltham, MA, USA) supplemented with 10% FBS (Sigma-Aldrich, Burlington, MA, USA). The TUBO cell line was derived from a mammary tumor arisen in a BALB-neuT female mouse and cultured in DMEM 20% FBS [45]. All cells tested negative for Mycoplasma using the MycoAlert kit (Lonza, Basilea, Switzerland), following the manufacturer’s instructions [46].

### 2.7. 3-(4,5-Dimethylthiazol-2-yl)-2,5-diphenyltetrazolium Bromide (MTT) Assay

5 × 10^3^ TUBO or 4T1 cells were cultured overnight in 96-well plates, and scalar doses of either Docetaxel, Ctrl_PLGA, or RGD_PLGA were then added. Viability was analyzed after 48 h using MTT. Briefly, MTT (0.5 mg/mL; Sigma Aldrich, Burlington, VT, USA) was added for 4 h at 37 °C, then supernatants removed and 150 μl/well dimethyl sulfoxide added to dissolve formazan crystals. Absorbance was measured on a 680XR microplate reader (BioRad Laboratories Inc., Hercules, CA, USA) at 570 and 650 nm (background subtraction) [47].

### 2.8. Flow Cytometric Analysis

To analyze integrin α_v_β_3_ expression, exponentially growing 4T1 or TUBO cells were detached, washed with washing buffer (PBS 0,2% BSA), and stained for 30 min at 4 °C with an anti-α_v_β_3_ integrin monoclonal antibody (cloneLM609, Millipore, Burlington, VT, USA), or with an isotype-matched negative control Ig. After washing, cells were stained with a carboxyfluorescein (FITC)-labelled rabbit anti-mouse Ig (Agilent Dako, Santa Clara, CA, USA) for 30 min at 4 °C [38]. To analyze the uptake of PLGA-NPs by BC cells, TUBO and 4T1 cells were incubated for 4 h at 37 °C with 12 μM Ctrl_PLGA and RGD_PLGA containing the FITC fluorochrome. At different time points, an aliquot of cells was harvested, washed, and analyzed using FACS for the FITC signal. To evaluate whether RGD_PLGA-NPs are internalized by the cells or remain bound to their surface, cells were incubated for 1 h with RGD_PLGA at 37 °C to allow internalization, or at 4 °C, a condition in which the internalization is impaired [48,49,50]. In parallel experiments, after washing, some samples were treated with acid pH (PBS 0.2% bovine serum albumin, 100 mM glycine, and 2 M urea (pH 2.5)) for 5 min at 4 °C to remove cell-surface-associated PLGA [48,51]. Cells were then washed and analyzed using FACS for the FITC signal [51]. In all samples, propidium iodide was added before acquisition in order to exclude dead cells from the analysis [52]. Cells were acquired on a BD FACSVerse (BD Bioscience, Franklin Lakes, NJ, USA) and analyzed using FlowJO10.5.3.

### 2.9. Confocal Microscopy Analysis of Cells

A total of 1.5 × 10^4^ 4T1 cells were seeded into 8-well μ-slides in fresh medium. 24 h later, cells were incubated with either Ctrl_PLGA or RGD_PLGA containing DOPE-CarboxyFluorescein for 30 min. Cells were then gently washed several times to remove any unbound PLGA-NPs. Z-stack images were acquired using laser scanning confocal microscopy (Leica TCS SP5, Leica Microsystems, Wetzlar, Germany) to obtain a series of parallel images along the Z-axis that could be used to reconstruct tridimensional images of the PLGA-labeled cells. The following wavelength was considered for microscopy: λ^ex^ = 495 nm, λ^em^ = 520 nm for DOPE-CF. Fluorescent images were processed using ImageJ Fiji freeware software.

### 2.10. Mice

Mice were bred and maintained under pathogen- and saprophytic-free conditions in the animal facility of the Molecular Biotechnology Center “Guido Tarone”, and treated in accordance with institutional and EU guidelines. The project was approved by the Animal Care and Use Committee of the University of Turin and by the Italian Ministry of Health (authorizations No. 107/2020-PR and 500/2017-PR).

1 × 10^4^ 4T1 cells were orthotopically injected into the third mammary gland of N = 40 8-week-old female BALB/c mice. For the pharmacokinetics experiments, mice were analyzed 20 days after cell injection, when bearing 200 mm^3^ mean volume tumors. For the therapeutic experiments, when the tumors reached 2 mm mean diameter, mice were blindly randomized into four groups, which received intraperitoneal (i.p.) injections of: the vehicle, i.e., 0.25 mL NaCl/HEPES solution (control); 5 mg/kg free Docetaxel; 5 mg/kg Ctrl_PLGA; 5 mg/kg RGD_PLGA. Mice were treated twice a week, for a total of 5 treatments. Mice were checked daily for any signs of suffering and weighed twice per week to assess pharmacotoxicity. Tumor diameters were measured twice per week with a caliper. One day after the last treatment, mice were sacrificed and tumors removed. Tumors were weighed, fixed in 4% formaldehyde, and processed for histology.

### 2.11. Magnetic Resonance Imaging of Phantom and Mice

MRI scans were acquired at 7 T using a Bruker Avance 300 spectrometer (Bruker, Billerica, MA, USA), equipped with the Micro 2.5 microimaging probe. MRI was carried out both on an in vitro phantom and on animals.

For in vitro analysis, a phantom was composed of glass capillaries filled with RGD_PLGA, Ctrl_PLGA or Gd-HPDO3A at variable concentrations.

T_2W_ images were acquired by using a standard RARE (rapid acquisition with refocused echoes) sequence with the following parameters: TR = 4000 ms, TE = 5.5 s, FOV = 10 mm × 10 mm, slice thickness = 1 mm, RARE factor = 32, matrix size = 128 × 128, in plane spatial resolution = 0.078 × 0.078 mm^2^/pixel, number of averages = 4.

T_1W_ images were acquired by using a standard MSME (multislice multiecho) sequence with the following parameters: TR = 250 ms, TE = 3.3 s, FOV = 10 mm × 10 mm, slice thickness = 1 mm, matrix size 128 × 128, in plane spatial resolution 0.078 × 0.078 mm^2^/pixel, number of averages = 6. ROIs were manually drawn.

The T_1_ contrast enhancement (T_1enh_%) was calculated as follows:

(5) $$T_{1\mathrm{enh}}\%=\frac{{\mathrm{SI}}_{\mathrm{sample}}-{\mathrm{SI}}_{\mathrm{ref}}}{{\mathrm{SI}}_{\mathrm{sample}}}\times 100$$

where SI_sample_ and SI_ref_ are signal intensities of the analyzed NP-sample and of the reference water-filled tube, respectively.

For in vivo analysis, tumor-bearing mice were anesthetized with 20 mg/kg tiletamine/zolazepam (Zoletil 100; Virbac SA, Carros, France) and 5 mg/kg xylazine (Rompun; Bayer, Leverkusen, Germany) by intramuscular injection using a 27-G syringe. MRI acquisitions were carried out before and after the i.v. injection of RGD_PLGA or Ctrl_PLGA (dose = 0.033 mmol/kg of Gd, 0.1 mL). A glass tube containing water was placed in proximity to the mouse, as MRI reference.

T_1W_ images were acquired by using a standard multislice multiecho (MSME) sequence, characterized by the following parameters: TR = 250 ms, TE = 3.3 s, FOV = 1 cm × 1 cm, slice thickness = 1 mm, matrix size = 128 × 128. T_2W_ images were acquired by using a standard rapid acquisition with refocused echoes (RARE) sequence with the following parameters: TR = 4000 ms, TE = 5.5 s, FOV = 30 mm × 30 mm, slice thickness = 1 mm, RARE factor = 32, matrix size = 128 × 128, in plane spatial resolution = 0.234 × 0.234 mm^2^/pixel, number of parallel slices = 9, number of averages = 4. ROIs were drawn manually.

The T_1_ contrast enhancement (T_1enh_%) was calculated as follows:

(6) $$T_{1\mathrm{enh}}\%=\frac{{\mathrm{SI}}_{\mathrm{post}}-{\mathrm{SI}}_{\mathrm{pre}}}{{\mathrm{SI}}_{\mathrm{pre}}}\times 100$$

where SI_post_ and SI_pre_ are signal intensities post- and pre-administration of the PLGA-NPs, normalized for signal in a reference water-filled tube.

Cardiac function was assessed by acquiring cardio-MR images with the same MRI scanner. A small animal ECG device (1025-MR, SA Instruments Inc., Stony Brook, NY, USA) was used for reliable electrocardiographic (ECG) synchronization and high-resolution heart imaging. Heart rate was monitored by electrocardiogram.

Many T_2w_ scout images were acquired in the transverse plane and the long axis plane of the left ventricle to determine the orientation of the short axis. The localization of the central short-axis slice was planned halfway between the apex and the base. The whole heart was covered by acquiring 5–7 short-axis parallel slices (slice thickness = 1 mm, number of slices was chosen to cover the entire heart). CINE MRI was carried out by using an ECG-triggered FLASH CINE gradient-spoiled gradient echo sequence with the following parameters: FA = 15°, TR = 8 ms, TE = 2.5 ms, FOV = 35 mm × 35 mm, matrix size = 192 × 192, in plane spatial resolution = 0.182 × 0.182 mm^2^/pixel, slice thickness = 1 mm, number of averages = 6, heart cycle sampled with 20 images. After the acquisition, morphological parameters of interest for cardiac function were measured in diastole and systole. Left end diastolic volume (EDV) and left end systolic volume (ESV) were manually measured. Then, left stroke volume (SV), cardiac output (CO) and ejection fraction (EF%) were calculated by applying the following formulae:

(7) SV=EDV−ESV

(8) $$\mathrm{EF}\%=\frac{\mathrm{SV}}{\mathrm{EDV}}\times 100$$

(9) CO=HR×SV

### 2.12. ICP-MS

At the end of the MRI experiments, the animals were sacrificed by cervical dislocation, in agreement with ethical rules. Cancer tissues, kidneys, liver, spleen, and muscles were explanted, weighed, and processed for ICP-MS analysis [53]. The tissues were added to 1 mL of concentrated HNO_3_ (70%). Upon dissolution of the tissues, samples were further digested through microwave heating (MicroSYNTH, Microwave labstation, Microsynth, Balgach, Switzerland, equipped with an optical fiber temperature control and HPR-1000/6M high-pressure reactor, Milestone, Bergamo, Italy). After digestion, ultrapure water was added to each sample up to a 2 mL total volume, then samples were filtered with 0.45 μm filter and analyzed using ICP-MS for quantification of Gd^3+^, using a Thermo Scientific ELEMENT 2 ICP-MS-Finnigan, Rodano, Italy. A calibration curve obtained by using four gadolinium absorption standard solutions (Sigma-Aldrich, Burlington, MA, USA) in the range 0.005–0.1 μg/mL was used for the quantification. The total mass of Gd^3+^ retained in each specimen was calculated with respect to the weight of the tumor tissue (as μg of Gd^3+^/g of tissue).

### 2.13. Histology

After MRI acquisition, mice were sacrificed. Cancer tissues, kidneys, liver, spleen, muscles, heart, and lungs were excised, washed twice in PBS, fixed in 5 in 4% formaldehyde and paraffin embedded. Sections of 5 μm thickness were cut with a microtome along the entire organ. Tumors were cut into 4 sections spaced by 50 µm. Sections were deparaffined with xylol and ethanol solutions, rinsed twice in PBS, and stained with hematoxylin/eosin (H/E stain) (BioOptic S.p.a., Milano, Italy). Samples were imaged under an Olympus BX41 microscope (Olympus, Tokio, Japan) equipped with a Leica photographic system (Leica Microsystems, Wetzlar, Germany).

### 2.14. Statistical Analysis

Statistical analyses were performed using the GraphPad8 software. All values are reported as means ± standard deviation or standard error. Data were analyzed using a two-tailed unpaired Student’s *t*-test, non-parametric Mann–Whitney or Kruskal–Wallis test, when the distribution calculated using a Shapiro–Wilk or Kolmogorov–Smirnov test was not normal. Differences in tumor growth were analyzed using two-way ANOVA with a Bonferroni post-test. *p* < 0.05 was considered significant.

## 3. Results

### 3.1. Preparation and Chemo-Physical Characterization of PLGA Nanoparticles

In this study, two types of PLGA-NPs were prepared and compared both in vitro and in vivo: (i) RGD-containing PLGA-NPs (hereinafter named RGD_PLGA) and (ii) control untargeted PLGA-NPs (hereinafter named Ctrl_PLGA).

The first batch of RGD_PLGA and Ctrl_PLGA contained an amphipathic Gd-complex (as an MRI probe), while the second batch contained Docetaxel (as a chemotherapeutic agent). All the nanoparticles also contained a fluorescent amphipathic probe (for optical microscopy) and a PEGylated phospholipid.

The general scheme of the NP formulation and chemical structures of the molecules used are shown in Figure 1A. The proposed NPs act as a stealth theranostic system, detectable by both optical imaging and MRI.

PLGA-NPs were prepared using the classic oil/water emulsion solvent extraction method [41]. Figure 1B reports chemicals used for PLGA-NP preparation, whereas Figure 1C reports the scheme of RGD binding.

All data regarding the PLGA-NPs’ formulations and their characterization are summarized in Table 1. RGD_PLGA and Ctrl_PLGA displayed a very high degree of analogy, from a chemo-physical perspective. The average hydrodynamic diameters, obtained by dynamic light scattering (DLS) measurements, corresponded to 146 ± 16 nm and 149 ± 12 nm for RGD_PLGA and Ctrl_PLGA, respectively (PDI < 0.1 for all preparations), with a negligible change in size upon RGD binding (Table 1). Analogously, only a slight difference in ζ-potential was recorded (−2.96 ± 0.3 mV vs. −3.69 ± 0.5 mV for RGD_PLGA and Ctrl_PLGA, respectively) (Table 1).

The amount of encapsulated Docetaxel was assessed using quantitative NMR. In fact, even though Docetaxel has a characteristic UV-vis absorption profile, the presence of the PLGA polymer (and other components) makes the background signal too intense and it is difficult to correctly extract the drug for its quantification. For this reason, Docetaxel quantification was carried out in NPs, without the Gd-complex, using high-resolution quantitative NMR, as reported in the Materials and Methods section. Data on NMR quantification have been added as Supplementary Materials (Figures S1–S3). The encapsulation efficiency (EE%) of Docetaxel was quantified as 18.2 ± 5 and 27.3 ± 7 for RGD_PLGA and Ctrl_PLGA, respectively. The lower EE% for RGD_PLGA is reasonably a consequence of the c(RGDfC) binding procedure and subsequent additional washing steps (Table 1).

**Figure 2:**
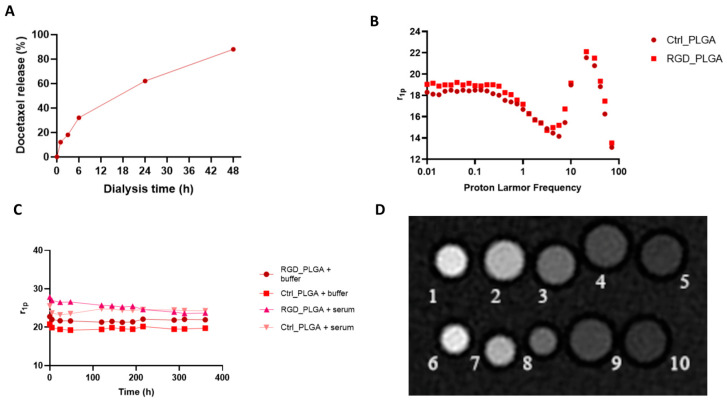
Chemo-physical characterization of PLGA nanoparticles. (**A**) In vitro release of Docetaxel. (**B**) NMRD profiles of Gd_PLGA-NPs. (**C**) Gd_PLGA-NPs’ molar relaxivities over time in HEPES/NaCl buffer and human serum for assessing PLGA stability over time. (**D**) Representative *T*_1w_ MR image of a phantom containing glass capillaries filled with variable concentrations of RGD_PLGA or Ctrl_PLGA (Legend: capillaries number 1–5 RGD_PLGA in PBS at variable concentrations, i.e., 1, 0.5, 0.2, 0.1, 0.05 mM; capillaries number 6–10 Ctrl_PLGA in PBS at variable concentrations, i.e., 1, 0.5, 0.2, 0.1, 0.05 mM).

The retention of the drug inside the NPs was assessed after multiple cycles of dialysis by NMR analysis of the external buffer (containing the eliminated Docetaxel). The results reported in Figure 2A indicate the analog kinetics of Docetaxel release from the two NPs, with no effect of c(RGDfC) on drug retention.

Subsequently, contrastographic and structural characterization of Gd-containing NPs was carried out using ^1^H-relaxometry (at fixed and variable magnetic field) and MRI at 7.1T. First, the Gd-DOTAMA encapsulation yield in PLGA was assessed by relaxometry (mineralization of the sample and relaxometric measurements, see Materials and Methods), demonstrating that it corresponded to 77% and 85% for RGD_PLGA and Ctrl_PLGA, respectively (Table 1). Hence, the number of Gd-DOTAMA molecules loaded in one nanoparticle (*ca*. 1 × 10^5^) is of the same order as that previously reported for other PLGA, liposomes or other NPs containing Gd-complexes (Table 1) [54,55].

The ^1^H-nuclear magnetic relaxation dispersion (NMRD) profiles of RGD_PLGA and Ctrl_PLGA were recorded at variable magnetic fields (0.01–80 MHz) and T = 25 °C (Figure 2B).

Relaxivities of the two PLGA-NP preparations were quite similar, with no significant contribution of c(RGDfC) addition. In the NPs’ NMRD profiles, a relaxivity hump at approximately 20–30 MHz was observed, typical of slowly moving systems (*r*_1p_ = 21–22 s^−1^mM^−1^ at B_0_ = 0.5 T, corresponding to a proton Larmor frequency of ca. 21 MHz). R.N. Mariano and co-workers reported an analog hump for Gd-DOTAMA-PLGA-NPs with a size of 140 nm [56]. They reported a strong reduction in the overall NMRD profile and of the hump when the NPs’ size increased up to the near disappearance of the contribution to relaxivity for micrometric PLGA particles [56]. Moreover, the relaxation enhancement has been suggested to be due to the strong interaction between the hydrophobic PLGA polymer and lipophilic chains of the Gd-complexes, which induces a reduction in the reorientational time (τ_R_) of the paramagnetic complex [56].

A relaxometric approach was also used to assess NP stability (Figure 2C). For this purpose, RGD_PLGA and Ctrl_PLGA were placed in isotonic HEPES/NaCl buffer or in the presence of human serum (Seronorm^TM^, Sero AS, Billingstad, Norway) at 37 °C for several days. Stability was checked by measuring the relaxation rates of NP suspensions at T = 37 °C and B_0_ = 0.5 T (corresponding to a proton Larmor frequency of 21 MHz). No significant change in the *r*_1p_ value was detected, indicating overall good stability of the NPs for at least 10 days. After this time, a small increase in the relaxation rate was observed for both NPs in buffer but not in serum. This suggests an overall small destabilization of PLGA-NPs due to hydrolysis of the polymer ester bonds. In the serum, stability was enhanced.

These data were corroborated by the assessment of no significant change in DLS size and ζ-potential in NPs on days after preparation. Moreover, these data indicate that serum albumin is not able to extract Gd-DOTAMA complexes from the PLGA-NP core, thus ensuring good stability in the blood for in vivo experiments.

Finally, the proof of concept of the feasibility of using such NPs as MRI contrast agents was carried out by acquiring MR images of a phantom composed of RGD_PLGA and Ctrl_PLGA at variable concentrations and comparing them with the standard clinically employed Gadoteridol (ProHance^®^, Bracco Imaging S.p.A., based on the Gd-HPDO3A complex). MRI showed a comparable enhancement of signal intensity for the two PLGA preparations, with or without RGD (Figure 2D). The good sensitivity of the Gd-DOTAMA incorporated into the PLGA-NPs is very promising for in vivo molecular imaging applications, where the accumulation of NPs in the tumor region is limited, and also in presence of a targeting system.

Altogether, the relaxometric measurements, DLS size, ζ-potential, and EE% clearly indicated that RGD_PLGA and Ctrl_PLGA share similar chemical/physical properties and a good stability profile over time, indicating possible applications for in vitro and in vivo experiments. Furthermore, MRI proof of concept indicated the feasibility of following NP biodistribution in vivo by MRI (see In vivo application of PLGA-NPs).

### 3.2. In Vitro Characterization of PLGA-NPs’ Binding to BC Cells

**Figure 3:**
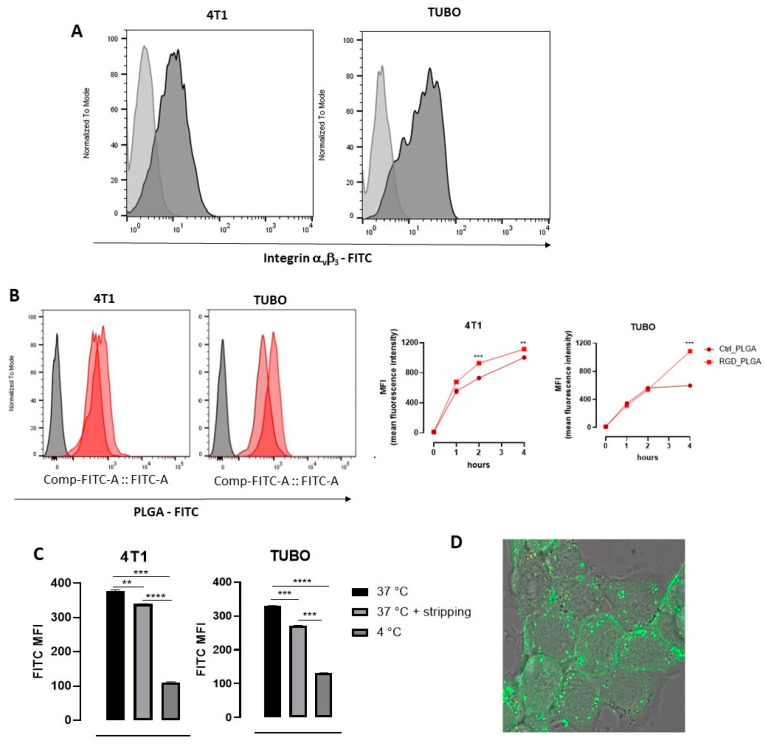
In vitro characterization of PLGA-NPs’ binding to BC cells. (**A**) Expression of α_v_β_3_ integrin on 4T1 and TUBO cell lines, measured by using a specific anti-α_v_β_3_ integrin monoclonal antibody. Representative FACS histograms are shown. Light gray histograms show the background signal obtained by staining cells with an isotype control antibody; dark gray histograms show the signal obtained by staining with the anti-α_v_β_3_ antibody. (**B**) FACS analysis of the kinetics of the uptake of PLGA-NPs in BC cells. 4T1 and TUBO cells were incubated at 37 °C with 12 μM FITC-containing Ctrl_PLGA or RGD_PLGA. Aliquots of cells were analyzed using FACS after 1, 2, and 4 h. Representative FACS histograms are shown, showing in gray the background signal of untreated cells, in light red the signal of cells incubated for 4 h with Ctrl_PLGA, and in dark red the histogram obtained in cells incubated for 4 h with RGD_PLGA. The graphs report the means ± SEM of the mean fluorescence intensity (MFI) measured in 2 independent experiments. (**C**) FACS analysis of RGD_PLGA-NP internalization into BC cells. 4T1 and TUBO cells were incubated for 1 h at 37 °C or 4 °C with 12 μM FITC-containing RGD_PLGA. An aliquot of cells was subjected to acidic stripping to remove membrane-bound PLGA-NPs before analysis. The graphs report the means ± SEM of the FITC MFI measured in two independent experiments. (**D**) Confocal fluorescence images of 4T1 cells upon 30 min incubation at 37 °C in presence of FITC-labelled RGD_PLGA-NPs. **, *p* < 0.01; ***, *p* < 0.001; ****, *p* < 0.0001, Student’s t test.

To assess the capability of RGD_PLGA and Ctrl_PLGA to deliver Docetaxel to BC cells in vitro, we first verified the expression of integrin α_v_β_3_ in BC cells. Triple negative 4T1 and HER2^+^ TUBO murine BC cell lines were stained with an anti-integrin α_v_β_3_ monoclonal antibody. Flow cytometric analysis showed that integrin α_v_β_3_ was expressed at high levels on the cell surface of both cell lines (Figure 3A), confirming our previous data obtained from TUBO cells [38].

4T1 and TUBO cells were then incubated with RGD_PLGA or Ctrl_PLGA ([Docetaxel] = 12 μM), which contained the FITC fluorochrome, and the fluorescent signal was analyzed using flow cytometry on aliquots of cells harvested after 1, 2 or 4 h. Incubation was performed at 37 °C to allow PLGA internalization in cells, since the PLGA-NP cell internalization mechanism was found to be mainly dependent on caveolae-mediated endocytosis [49], and RGD can induce integrin receptor-mediated endocytosis, which are both active at 37 °C but impaired at 4 °C [50]. As shown in Figure 3B, the fluorescent signal progressively increased in 4T1 and TUBO cells treated with both compounds. However, at 4 h, TUBO cells displayed a lower uptake of Ctrl_PLGA than 4T1 cells, likely reflecting different degrees of endocytosis in the two cell lines. Notably, in both cell lines, cells incubated with RGD_PLGA showed a higher signal than those treated with Ctrl_PLGA, and the levels measured at 4 h were similar in 4T1 and TUBO cells, indicating that RGD increases the delivery of the PLGA to BC cells and may overcome a cell’s intrinsic low endocytosis of untargeted PLGA-NPs.

To verify whether RGD_PLGA-NPs were internalized by the cells or remained bound to their surface, cells were incubated for 1 h with RGD_PLGA at 4 or 37 °C, then PLGA bound to the cell membrane were stripped by incubation with an acidic solution, and cells were analyzed using flow cytometry. As shown in Figure 3C, cells incubated with RGD_PLGA at 37 °C showed a significantly higher FITC signal than cells incubated with RGD_PLGA at 4 °C, suggesting that RGD_PLGA was internalized in cells. Indeed, when cells incubated at 37 °C were treated with an acidic solution to remove all the PLGA bound to the cell surface, only a mild reduction in the signal was observed, confirming that the majority of RGD_PLGA was internalized by BC cells.

The internalization of RGD_PLGA inside the cells was further confirmed by confocal fluorescence microscopy of 4T1 cells incubated for 30 min at 37 °C in presence of FITC-labelled RGD_PLGA-NPs (Figure 3D). The fluorescence signal localized in discrete spots clearly indicated the internalization of PLGA-NPs and compartmentalization in endosome-like vesicles.

### 3.3. Assessment of BC Cells Viability upon Treatment with Docetaxel Loaded PLGA Nanoparticles

**Figure 4:**
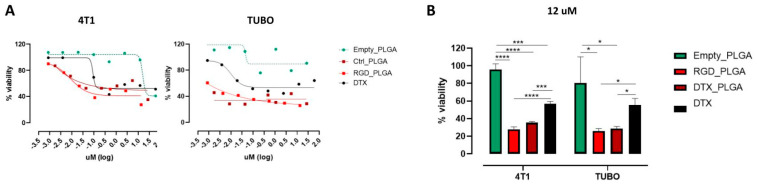
Effect of DTX_PLGA on BC cell viability. (**A**) MTT assays on TUBO and 4T1 cell lines performed with PLGA-NPs with and without Docetaxel, and Docetaxel alone at various concentrations. (**B**) Cell viability of the same cells treated with 12 μM Docetaxel (free or encapsulated in PLGA-NPs), or with empty PLGA. *, *p* < 0.05; ***, *p* < 0.001; ****, *p* < 0.0001, Student’s *t*-test.

To analyze whether RGD_PLGA and Ctrl_PLGA-NPs loaded with Docetaxel could affect the viability of BC cells and improve the efficacy of Docetaxel, 4T1 and TUBO cells were treated for 48 h with scalar doses of free Docetaxel, RGD_PLGA or Ctrl_PLGA containing Docetaxel, or with empty PLGA as a control. Cell viability was measured using an MTT assay. As shown in Figure 4A, empty PLGA did not exert a toxic effect on 4T1 or TUBO cells, and only the highest dose of PLGA slightly impaired their viability. In contrast, both RGD_PLGA and Ctrl_PLGA significantly impaired the viability of 4T1 and TUBO cells, and their effects were greater than those exerted by free Docetaxel. Statistical analysis of the results obtained with 12 μM Docetaxel demonstrated that RGD_PLGA and Ctrl_PLGA induced a significantly higher reduction in cell viability than Docetaxel alone (Figure 4B).

### 3.4. In Vivo Application of PLGA-NPs and Biodistribution

Based on the in vitro results, experiments using mouse models of BC were performed. For this purpose, 1 × 10^4^ 4T1 TNBC cells were injected into the adipose tissue of the third mammary gland of syngeneic BALB/c female mice. After 20 days, the tumors reached a mean volume of ca. 200 mm^3^, which was considered suitable for clinical applications. First, the in vivo biodistribution of RGD_PLGA and Ctrl_PLGA was assessed by MRI. Mice bearing 4T1 were treated with Gd-DOTAMA-containing RGD_PLGA or Ctrl_PLGA, administered intravenously at a dose of 0.033 mmol/kg of Gd. This dose of Gd is ca. twenty-fold lower than the minimum dose normally used for clinical application (in clinical MRI 0.05–0.4 mmol Gd/kg are used for humans, corresponding to 0.6–4.8 mmol/kg in mice) [57].

Mice were subjected to MRI (Bruker 7.1 T spectrometer) before the administration of RGD_PLGA or Ctrl_PLGA and at t = 15 min, t = 4 h and t = 24 h post injection. Anatomical *T*_2w_ and Gd-contrasted *T*_1w_ scans were acquired. Signal enhancement in *T*_1w_ images after the injection of NPs was calculated with reference to pre-injection images for the tumor region, muscles, spleen, liver, and kidneys.

**Figure 5:**
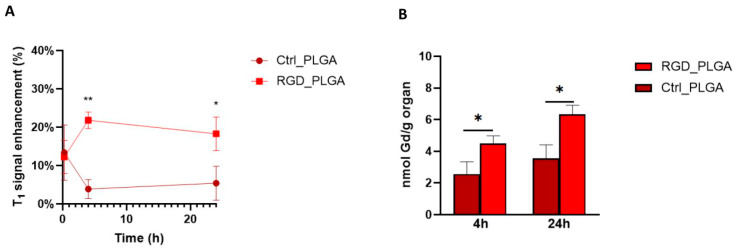
In vivo biodistribution of RGD_PLGA and Ctrl_PLGA. (**A**) *T*_1_^enh^ in the tumor region at different time points upon administration of RGD_PLGA or Ctrl_PLGA. (**B**) Quantification of Gd retained in the tumor by inductively coupled plasma mass spectrometry (ICP-MS), at t = 4 h and t = 24 h. *, *p* < 0.05; **, *p* < 0.01, Student’s *t*-test.

*T*_1w_ MR images of the tumor area were analyzed at different time points, and data are shown in Figure 5. In the tumor region, *T*_1enh_ was ca. 12% for both applications of RGD_PLGA or Ctrl_PLGA because of the passive accumulation of PLGA-NPs in the tumor region through the enhanced and permeability retention (EPR) effect. This value decreased to less than 5% at t = 4 h and t = 24 h for Ctrl_PLGA, owing to the natural *wash-out* effect. Conversely, in mice treated with RGD_PLGA, *T*_1_^enh^ increased to ~22% at t = 4 h and was maintained to ~18% at t = 24 h (Figure 5A). This is a consequence of active targeting and accumulation in the integrin-rich tumor region. These data are in line with data from the literature, showing that RGD-targeting normally has an accumulation peak at approximately 3–6 h post injection [36,37]. MRI analysis of other organs showed that the elimination of PLGA-NPs occurs through the liver route, as generally occurs for nanosystems [25]. In fact, *T*_1enh_ in the liver was ~21% for both RGD_PLGA and Ctrl_PLGA at t = 15 min, then it increased up to 38% and 55% for RGD_PLGA at t = 4 h and 24 h and up to 29% and 37% for Ctrl_PLGA at t = 4 h and 24 h, respectively (Figure S4). Analysis of the muscle and kidney revealed only a small signal enhancement (lower than 10%) for both NPs at all tested time points (Figure S4). Finally, in the spleen, there was a higher accumulation of RGD_PLGA at t = 15 min and t = 4 h than in Ctrl_PLGA. At t = 24 h, no significant difference in signal enhancement in the spleen was revealed by comparing RGD_PLGA and Ctrl_PLGA.

The higher accumulation of targeted PLGA-NPs in the liver and spleen may be due to the expression of integrins in macrophages, which are abundant in these organs [40].

After MRI analysis, tumors and other organs were excised for analytical quantification of Gd retained in the organs by ICP-MS, at t = 4 h and t = 24 h. Figure 5B and Figure S5 report the quantification of Gd in the tumor and liver, respectively. Analytical data confirmed the MRI biodistribution. At both t = 4 h and t = 24 h, the accumulation of Gd was more intense in both the tumors and liver of mice treated with RGD_PLGA than in mice treated with Ctrl_PLGA (approximately 44% and 48% higher in the two organs, respectively). Similar to what was demonstrated by MRI, the accumulation in the liver was much higher than that in the tumors, as expected for all drugs, and especially for nanosystems. Quantification of the muscles, kidneys and spleen is shown in Figure S5. As for MRI, a low Gd concentration was reported in the muscles and kidneys.

MRI experiments and ICP analysis reported a good effect of using PLGA-NPs, which were able to accumulate in the tumor region. This effect was more pronounced in the presence of RGD.

Using quantitative ICP-MS analysis, it was estimated that ca. 2.0 × 10^16^ and 3.1 × 10^16^ RGD_PLGA-NPs per gram of tumor tissue can be accumulated at t = 4 h and t = 24 h, respectively.

Hence, the use of RGD_PLGA can increase the effective dose in the tumor, minimizing side effects on distal organs.

### 3.5. In Vivo Application for Anticancer Therapy

**Figure 6:**
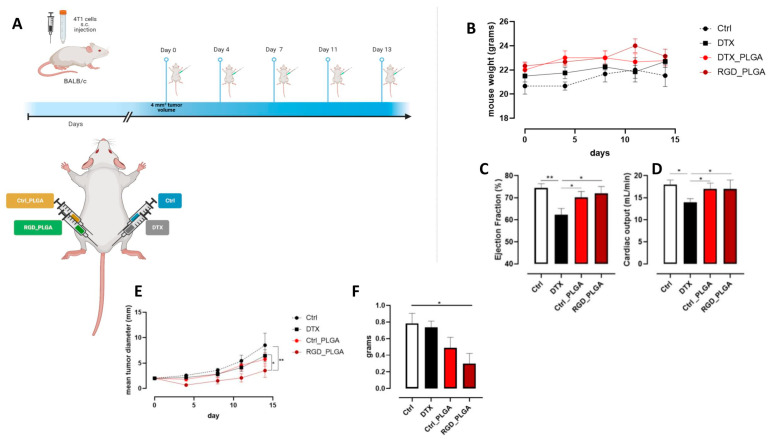
In vivo effect of PLGA-NPs. (**A**) BALB/c mice were orthotopically challenged with 1 × 10^4^ 4T1 cells. When the tumors reached 2 mm in diameter, the mice were treated with RGD_PLGA, Ctrl_PLGA, free docetaxel, or physiological saline solution as a control (N = 6 per group). The treatment was repeated twice per week for a total of 5 administrations. Graphs show (**B**) mouse weight, (**C**) heart ejection fraction (EF%) as assessed by cardioMRI, (**D**) heart cardiac output (CO) as assessed by flash cine cardioMRI, (**E**) mean tumor diameter, and (**F**) tumor weight at the end of the experiment. *, *p* < 0.05; **, *p* < 0.01; two-way ANOVA (**F**), unpaired Student’s t test (**C**,**D**) or Mann–Whitney test (**E**). Panel A was created with Biorender.com.

To analyze the therapeutic efficacy of the NPs, mice were treated with RGD_PLGA or Ctrl_PLGA. 4T1 cells were injected into the third mammary gland of female BALB/c mice. When the tumors reached a mean diameter of 2 mm, a dimension that we know to be suitable for therapeutic studies [45,46,58,59,60], tumor-bearing mice were randomly assigned to four different experimental groups, treated i.p. as follows: (i) RGD_PLGA; (ii) Ctrl_PLGA; (iii) free docetaxel, as a control; (iv) physiological saline solution. Figure 6A shows the treatment protocol used. The mice were treated twice per week for a total of five treatments. A total dose of 25 mg/Kg of docetaxel was administered to each mouse. At this dose, docetaxel, either free or encapsulated into PLGA-NPs, did not induce systemic toxicity, as demonstrated by the lack of reduction in body weight in treated mice compared to the controls (Figure 6B).

Since docetaxel is known to have negative side effects on heart function [7,8], cardio MR images were acquired for the four groups of mice. For this purpose, cine FLASH cardio MRI was recorded and EF% and CO were measured as previously reported [8].

In Figure S6, systolic and diastolic MR images of the heart regions for representative mice of the four groups are shown. Quantitative data for EF% and CO are shown in Figure 6C,D, respectively. In mice treated with free docetaxel, a significant decrease in the two cardiac parameters was observed compared to that in control mice (EF% = 62% vs. 75%; CO = 14 mL/min vs. 18 mL/min for mice treated with docetaxel and control mice, respectively). Treatment with NPs (both RGD_PLGA and Ctrl_PLGA) preserved the cardiac function (EF% = 72% and CO = 17 mL/min in RGD_PLGA-treated mice; EF% = 70% and CO = 17 mL/min in Ctrl_PLGA-treated mice). This effect was present for both PLGA formulations because it was not due to the presence of the active targeting moiety but to the caging of docetaxel inside PLGA and the active/passive accumulation in the tumor region.

Treatment with free docetaxel did not hinder tumor growth, as reported in previous studies using similar amounts of docetaxel in 4T1-tumor-bearing mice [61,62], while Ctrl_PLGA induced a mild, not significant, effect. In contrast, treatment with RGD_PLGA significantly slowed tumor growth compared to that in control mice (Figure 6E). Thus, tumors explanted from RGD_PLGA-treated mice displayed a significantly lower weight than those explanted from control or free docetaxel-treated mice (Figure 6F), indicating that RGD_PLGA increased the efficacy of docetaxel in vivo.

**Figure 7:**
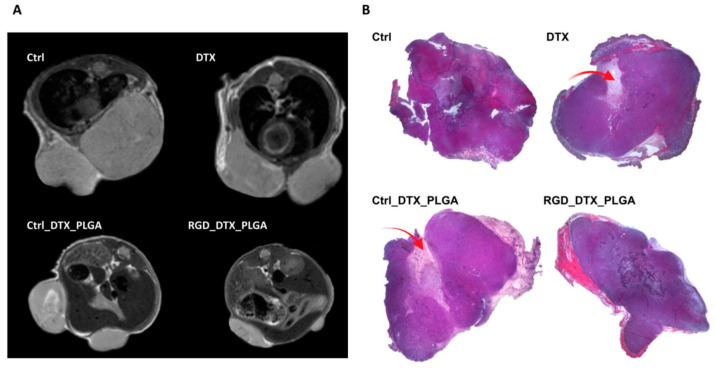
MRI and histological analyses of 4T1 tumors from mice treated with free docetaxel or with PLGA-NPs containing docetaxel. (**A**) Representative axial *T*_2w_-MR images of tumor region in mice treated with physiological saline solution as a control, free docetaxel (DTX), Ctrl_PLGA or RGD_PLGA (N = 6 per group). (**B**) Representative hematoxylin/eosin histological staining of 4T1 tumors of mice belonging to the four groups of treatment. The red arrows highlight the necrotic areas.

Finally, MRI was carried out at the treatment end point. Representative axial *T*_2w_-MR images of tumors at the endpoint are reported in Figure 7A. MRI confirmed data obtained by caliper measurements. MR images showed that tumors are heterogeneous, with the onset of necrotic area, especially in mice treated with free docetaxel. Both RGD_PLGA and Ctrl_PLGA provided a significant decrease in tumor growth, with a more intense effect of targeted PLGA. Figure 7B reports representative hematoxylin/eosin staining of tumors, confirming the lower size of tumors in mice treated with RGD_PLGA with respect to the other groups. Furthermore, mice treated with RGD_PLGA show reduced areas of necrosis with respect to control mice and mice treated with free docetaxel or with Ctrl_PLGA.

## 4. Discussion

The treatment of BC remains a challenging task. Indeed, targeted therapies are available only for a small proportion of patients affected by specific BC subtypes, such as HER2^+^. Generally, most BC patients still rely on surgery, chemo- and radiotherapy [1]. In particular, taxane-based chemotherapy is widely used for advanced BC treatment.

Taxanes display poor aqueous solubility, which is currently improved by adding large amounts of nonionic surfactants to the formulations used in the clinic. However, these surfactants may have considerable side effects [63]. In this scenario, we decided to design a nanosystem for the delivery of antitumor drugs that could improve their therapeutic efficacy and reduce their side effects.

Several nanosystems, including virus-like particles, liposomes, and silica-based NPs, have been developed to deliver chemotherapy. However, these systems are immunogenic and display slow degradation in vivo [64]. To avoid these drawbacks, PLGA-NPs were selected in this study because they are one of the most attractive biocompatible and biodegradable nanocarriers in current clinical and preclinical practices [11]. More than twenty PLGA-based drug products have been approved by the FDA, in which the PLGA provides sustained drug release in long-acting formulations such as NPs and solid or gel implants [65]. PLGA-NPs are characterized by a hydrophobic core, allowing the entrapping of hydrophobic molecules which are water-insoluble, such as docetaxel, a cornerstone in BC treatment. Docetaxel is a microtubule inhibitor, capable of interfering with cancer cell division and eventually causing cell death via apoptosis [30]. Encapsulation of docetaxel in PLGA has already been performed and tested in different preclinical models of cancer, with different NP formulation or functionalizations, reporting generally positive results [28,66,67,68,69]. However, to improve tumor targeting, we decided to functionalize PLGA with the RGD peptide for active targeting of α_v_β_3_ integrins, which are overexpressed in BC cells [38]. Recently, RGD was successfully exploited to deliver PLGA–poloxamer NPs containing an inhibitory neuropeptide to HeLa cells [70]. Similarly, RGD_PLGA has been used to target HUVEC and transplantable liver cancer cells [35].

To assess whether RGD could improve PLGA targeting to BC and ameliorate docetaxel-based therapy, two different classes of PLGA-NPs, untargeted (Ctrl_PLGA) and RGD-functionalized (RGD_PLGA), were prepared using oil-in-water emulsion and compared in both in vitro and in vivo BC models. In parallel experiments, both targeted and untargeted NPs were formulated to load docetaxel or an amphipathic Gd-complex, exploited for NP characterization and to follow their in vivo biodistribution and accumulation. Indeed, the above-described PLGA-NPs are versatile systems, since they can work either as MRI probes or as optical probes, depending on the formulation, including Gd-complexes or FITC. All NPs also held at least one PEGylated phospholipid, used for multiple reasons, e.g., to improve NPs’ suspensibility and reduce their aggregation, to limit their uptake by macrophages, enhance their blood half-life, and prevent nonspecific interactions with cellular surfaces. It is well known that PEG chains create a protective hydrophilic layer around the NPs, thus reducing serum-dependent phagocytosis by inhibiting opsonin protein adsorption on NP surfaces [25]. The prepared PLGA-NPs had a diameter of approximately 150 nm and a small negative charge on the surface, with no significant inhomogeneity resulting from the RGD ligation. The amount of encapsulated docetaxel, measured through NMR to circumvent PLGA-related background signals in UV/vis spectrometry, corresponds to ca. 18% and 27% of the total for RGD_PLGA and Ctrl_PLGA, respectively. The lower EE% value for RGD_PLGA is reasonably because of the cyclic RGD ligation and subsequent additional washing steps. The water proton longitudinal relaxation rate (*R*_1_) values indicate that the NPs are highly stable under physiological conditions over a 10-day period. Experiments carried out on TN 4T1 and HER2^+^ TUBO cells demonstrated that PLGA-NPs successfully reached the BC cell surface, especially with the RGD-targeting moiety. RGD_PLGAs could also penetrate the cells, as assessed through flow cytometry and confocal microscopy. It is important to note that the addition of the RGD moiety was able to restore the lower level of PLGA endocytosis shown by TUBO cells than 4T1 cells, which suggests that RGD_PLGA may be successfully used for drug delivery in tumors that show a low endocytic capacity. The mechanisms underlying the intracellular differences affecting NP cell uptake remain to be elucidated, although endocytic profiling of different cancer cell lines has recently identified differences in the organization of both the endolysosomal and the Golgi systems that influence membrane trafficking between the plasma membrane and endosomes, determining the rate of untargeted PLGA internalization [71].

In vitro analysis showed that treatment with DTX_PLGA was more effective in inducing TUBO and 4T1 cytotoxicity than docetaxel alone, whereas the empty PLGA exhibited no toxic effects. This result shows that the observed pharmacological effect is not related to the NPs’ structure, confirming the biocompatibility and nontoxicity of this polymer. Similar results were obtained in human MCF-7 and MDA-MB-231 BC cells using docetaxel-loaded PLGA containing d-α-tocopherol polyethylene glycol 1000 succinate (TPGS) as a surfactant [66], while other studies did not show significant differences in the viability of MDA-MB-231 cells treated with either free docetaxel or rod-shaped docetaxel-loaded PLGA-NPs, suggesting that PLGA efficacy can vary according to the NP formulation [28].

Altogether, the nice docetaxel entrapment and the successful delivery achieved by the NPs persuaded us to proceed with their in vivo administration. For this purpose, we used BALB/c female mice that received orthotopic 4T1 injections to induce the development of TNBC. MR images revealed good accumulation of PLGA-NPs at the lesion site, owing to the EPR effect. As expected, the presence of the RGD moiety enhanced tumor accumulation of the PLGA nanocarriers, especially in the first hours after injection. Interestingly, data in the literature indicate that RGD-mediated targeting leads to a peak in NP accumulation approximately 3–6 h after injection, as we previously demonstrated using an RGD-based optical imaging probe [34]. Accumulation was also confirmed by ICP-MS, strengthening our assumption of using PLGA-NPs to help the drug selectively localize at the tumor site, with the aim of reducing drug doses and, hence, unwanted effects on distal organs. Indeed, α_v_β_3_ integrin is not only overexpressed in BC cells but also in the tumor endothelium [72]. RGD_PLGA-NPs may, thus, extravasate more effectively than untargeted PLGA-NPs, and enter the tumor owing to the enhanced permeability and retention effect. This increased accumulation in the tumor allows the RGD peptide to bind to α_v_β_3_ integrins expressed in BC cells, inducing their cellular uptake and drug delivery.

Through therapeutic experiments in this BC murine model, carried out by administering Ctrl_PLGA or RGD_PLGA five times over a 2-week period, for a total dose of 25 mg/kg of docetaxel per mouse, we demonstrated an increased therapeutic efficacy of Ctrl_PLGA with respect to free docetaxel, and even higher improvements were obtained with RGD_PLGA. Indeed, tumors from mice treated with RGD_PLGA underwent enhanced growth reduction and appeared shrunken and less necrotized. Notably, free docetaxel did not significantly impair tumor growth, in accordance with previous studies reporting no effects of treatment of 4T1-tumor-bearing mice with similar doses of docetaxel [61,62]. To obtain an anticancer effect in this tumor model, higher doses of free docetaxel should be used, which increases the probability of developing side effects [73,74]. Finally, we monitored cardiac parameters (EF% and CO) to check heart-related side effects of the treatment, demonstrating that the entrapment of Docetaxel in the PLGA was significantly less harmful to cardiac function.

Summing up, the obtained results demonstrated that RGD_PLGA-NPs are stable and nontoxic nanocarriers, endowed with higher efficiency in hindering cancer cell viability compared to untargeted PLGA and to free Docetaxel. Thus, exploitation of the RGD-targeting system was a winning choice throughout the entire set of experiments, significantly improving drug delivery. Notably, the administration of Docetaxel encapsulated into NPs reduced its deposition in distal organs (e.g., heart), consequently lowering the side effects. These promising data suggest that PLGA-NPs have great potential for use in BC therapies.

## 5. Conclusions

In this study, we developed a Docetaxel-loaded PLGA-NP system functionalized with an RGD tripeptide to improve BC targeting. In vitro functional experiments showed that the use of these NPs induced higher antitumor activity than free Docetaxel or untargeted PLGA-NPs. In vivo studies demonstrated that RGD_PLGA-NPs enhanced drug accumulation in the tumor, significantly impairing tumor growth. Thus, this study demonstrated the possibility of improving the efficacy of PLGA-NPs as a nanosystem for specific drug delivery in BC by the addition of the RGD peptide. RGD_PLGA-NP-based drug delivery may provide a strategy for overcoming resistance and increasing drug concentrations in tumor cells and the tumor microenvironment in patients with drug-resistant BC. Thus, we have very recently started to put efforts into exploiting these nanosystems for combined therapy, involving both a chemotherapeutic agent and small molecules targeting tumor antigens that play important roles in tumor progression. These PLGA-NP-based therapies are valuable means for the development of novel strategies for BC treatment, and further investigation can provide groundbreaking advances in the next few years, possibly leading to their clinical application.

## Figures and Tables

**Table 1:** PLGA-NPs employed in this work.

	RGD_PLGA		Ctrl_PLGA		
Name	RGD_PLGA	RGD_PLGA	Empty_PLGA	Ctrl_PLGA	Ctrl_PLGA
Formulation	PLGA, DOPE-CF, DSPE-PEG2000 maleimide, DPPE-PEG2000-methoxy, Gd-DOTAMA, c(RGDfC).	PLGA, DOPE-CF, DSPE-PEG2000-maleimide, DPPE-PEG2000-methoxy, DTX, c(RGDfC).	PLGA, DOPE-CF, DPPE-PEG2000-methoxy, c(RGDfC).	PLGA, DOPE-CF, DPPE-PEG2000-methoxy, Gd-DOTAMA, c(RGDfC).	PLGA, DOPE-CF, DPPE-PEG2000-methoxy, DTX, c(RGDfC).
Hydrodynamic diameter (nm) + PDI measured by DLS at 25 °C in HEPES/NaCl buffer	135 PDI = 0.10	158 PDI = 0.07	149 PDI = 0.08	161 PDI = 0.09	136 PDI = 0.07
ζ-potential (mV) measured by DLS at 25 °C in HEPES/NaCl buffer	−2.59	−3.32	/	−2.52	−4.85
Relaxivity (mM^−1^ s ^−1^) at 21.5 MHz and 25 °C	24.10	/	/	24.76	/
Gd-DOTAMA (%)	77 ± 5%	/	/	85 ± 8%	/
Number of Gd/NP	1.2 ± 0.1 × 10^5^	/	/	1.3 ± 0.1 × 10^5^	/
Docetaxel encapsulation efficiency (EE%)	/	18.2 ± 5	/	/	27.3 ± 7

## Data Availability

The data presented in this study are available in the article and Supplementary Materials.

## Supplementary Materials

The following supporting information can be downloaded at: https://www.mdpi.com/article/10.3390/cancers15010008/s1. Figure S1. NMR spectra of Docetaxel; Figure S2. Docetaxel calibration curve; Figure S3. NMR spectra of PLGA-NPs; Figure S4. MRI biodistribution of Ctrl_PLGA or RGD_PLGA-NPs; Figure S5. ICP-MS quantification of Gd in organs upon administration of Ctrl_PLGA or RGD_PLGA-NPs; Figure S6. Cardio-MR images.
