# Supplementary material for: RGD_PLGA Nanoparticles with Docetaxel: A Route for Improving Drug Efficiency and Reducing Toxicity in Breast Cancer Treatment
Source: Cancers (Basel). 2022 Dec 20;15(1):8. doi: 10.3390/cancers15010008 (PMC9817983; doi:10.3390/cancers15010008)
Supplement: Supplementary file 1 [file cancers-15-00008-s001.zip › cancers-2059064-supplementary.pdf]

Supplementary material

RGD_PLGA Nanoparticles with Docetaxel: A Route for Improving Drug Efficiency and Reducing Toxicity in Breast Cancer Treatment

Enza Di Gregorio *,†, Chiara Romiti †, Antonino Di Lorenzo *, Federica Cavallo, Giuseppe Ferrauto ‡ and Laura Conti ‡

Department of Molecular Biotechnology and Health Sciences, University of Turin, Via Nizza 52, 10126 Turin, Italy

* Correspondence: enza.digregorio@unito.it (E.D.G.); antonino.dilorenzo@unito.it (A.D.L.); Tel.: +39 011-6708459 (E.D.G.); +39 011-6706458 (A.D.L.)

† These authors contributed equally to this work.

‡ These authors contributed equally to this work.

Keywords: breast cancer; Docetaxel; PLGA nanoparticles; magnetic resonance imaging; RGD; theranostic

Supplementary figures

Figure S1. NMR spectra of Docetaxel

Figure S2. Docetaxel calibration curve

Figure S3. NMR spectra of PLGA NPs.

Figure S4. MRI biodistribution of Ctrl_PLGA or RGD_PLGA NPs

Figure S5. ICP-MS quantification of Gd in organs upon administration of Ctrl_PLGA or RGD_PLGA NPs

Figure S6. Cardio-MR images

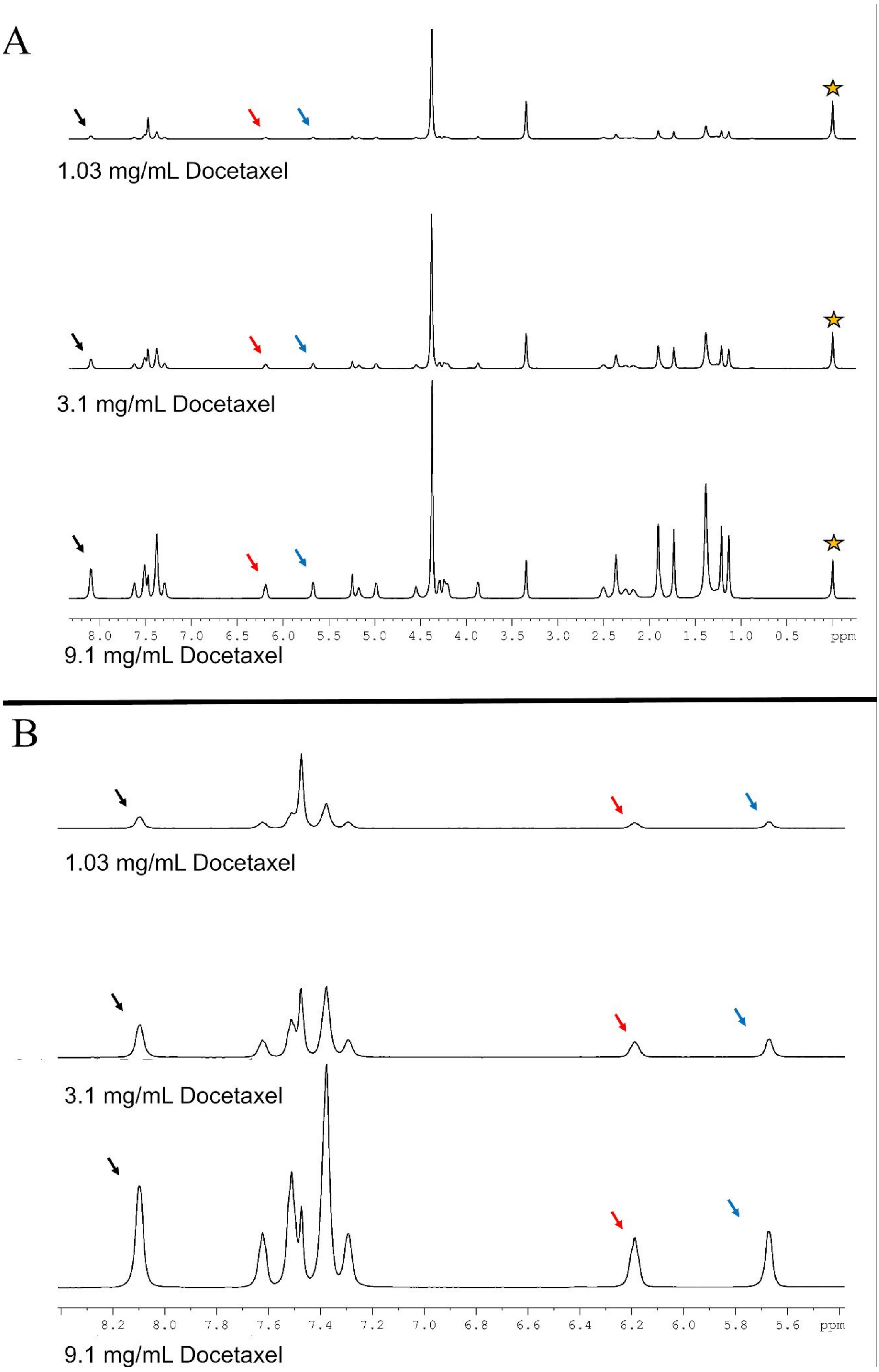

Figure S1. (A) NMR spectra of Docetaxel at variable concentration in CDCl3/MetOD (2:1 vol/vol) in presence of 0.33 mg/mL standard TSP) (B_0==14 T, T=298K). (B) magnification of spectra reported in (A). Yellow stars indicate NMR resonance of TSP (3-Trimethylsilylpropanoic acid) standard; black, red and blue arrows indicate characteristic resonances of Docetaxel used for calibration curves reported in Figure S3 (8.1, 6.19, 5.68 ppm, respectively).

	Int. 8.1	Int. 6.19	Int. 5.68
Best-fit values			
Slope	0.06576	0.06655	0.06760
Y-intercept	-0.004045	-0.002897	-0.005842
X-intercept	0.06152	0.04353	0.08643
1/slope	15.21	15.03	14.79
Std. Error			
Slope	0.0009047	0.001185	0.001560
Y-intercept	0.004459	0.005842	0.007688
95% Confidence Intervals			
Slope	0.06187 to 0.06965	0.06145 to 0.07165	0.06089 to 0.07431
Y-intercept	-0.02323 to 0.01514	-0.02803 to 0.02224	-0.03892 to 0.02724
X-intercept	-0.2401 to 0.3399	-0.3531 to 0.4010	-0.4332 to 0.5409
Goodness of Fit			
R square	0.9996	0.9994	0.9989
Sy.x	0.006528	0.008553	0.01126
Is slope significantly non-zero?			
F	5284	3152	1878
DFn, DFd	1, 2	1, 2	1, 2
P value	0.0002	0.0003	0.0005
Deviation from zero?	Significant	Significant	Significant
Equation	Y = 0.06576*X - 0.004045	Y = 0.06655*X - 0.002897	Y = 0.06760*X - 0.005842

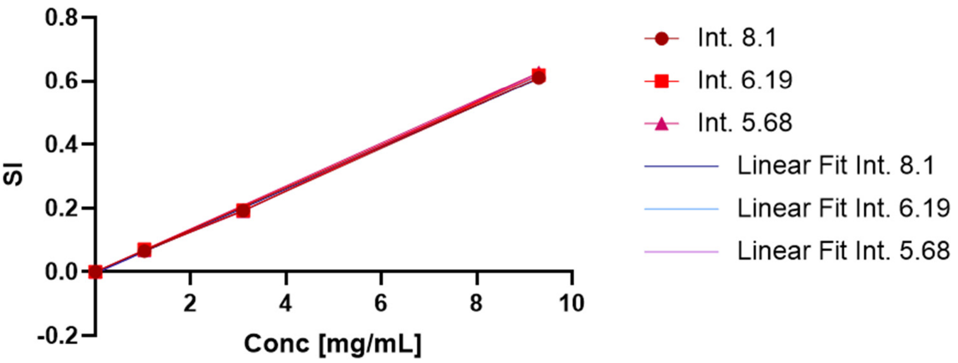

Figure S2. Docetaxel calibration curve obtained through NMR spectroscopy (spectra in Fig.S2)

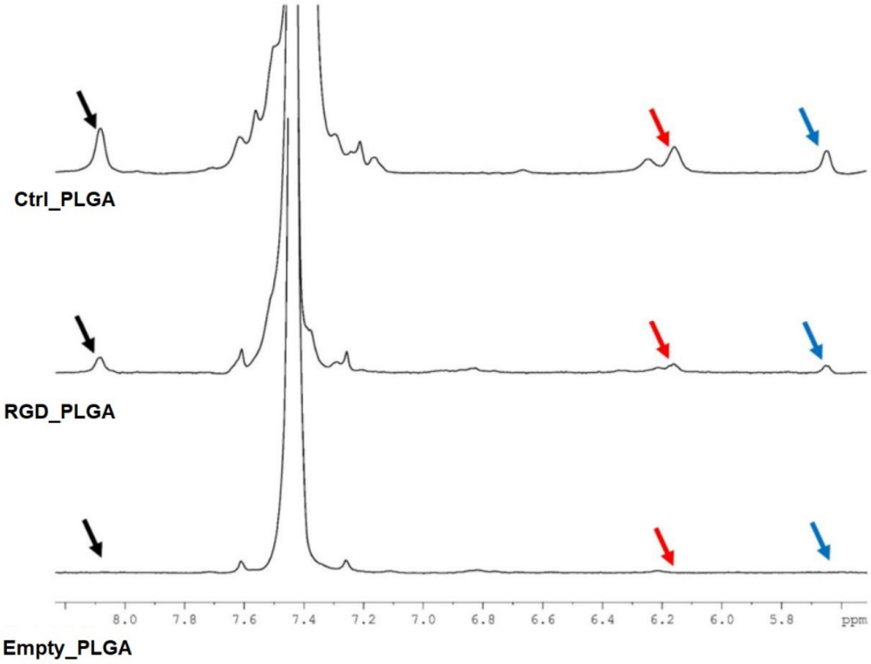

Figure S3. NMR spectra of PLGA NPs in CDCl3/MetOD (2:1 vol/vol) in presence of 0.33 mg/mL standard TSP) (B0==14 T, T=298K) normalised respect to the signal of standard TSP reference. Black, red and blue arrows indicate characteristic resonances of Docetaxel (8.1, 6.19, 5.68 ppm, respectively), used for the quantification of the drug content using the calibration curves reported in Figure S3.

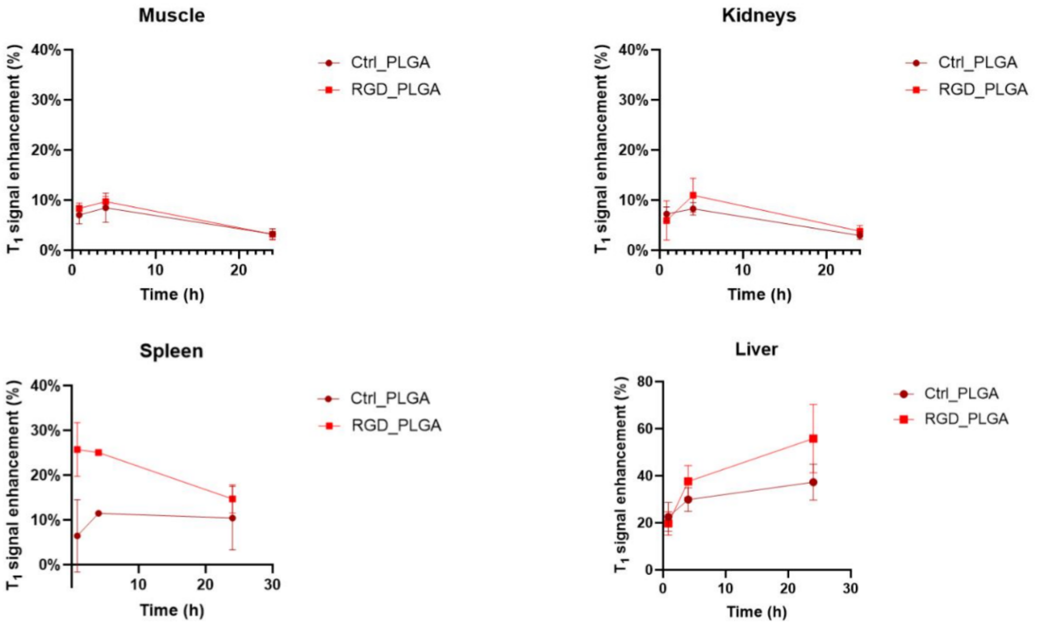

Figure S4. ***T*₁ MRI signal enhancement (%) in muscles, kidneys, spleen, and liver upon i.p. administration of RGD_PLGA or Ctrl_PLGA at t= 5min, 4h or 24h.**

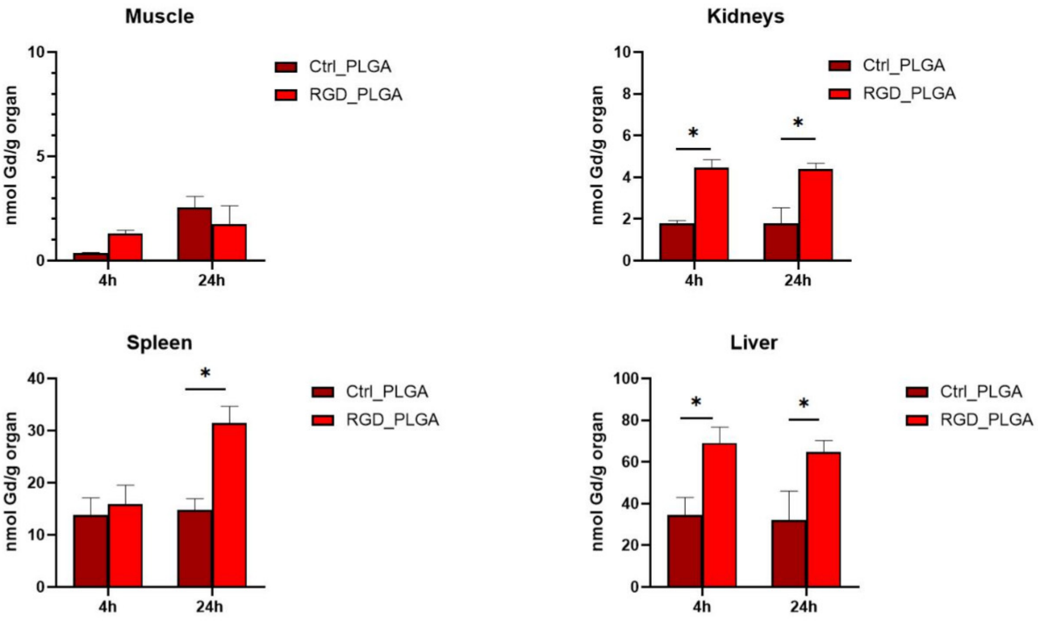

Figure S5. Quantification of Gd by ICP-MS in muscles, kidneys, spleen, and liver upon i.v. administration of RGD_PLGA or Ctrl _PLGA at t=4h or 24h.

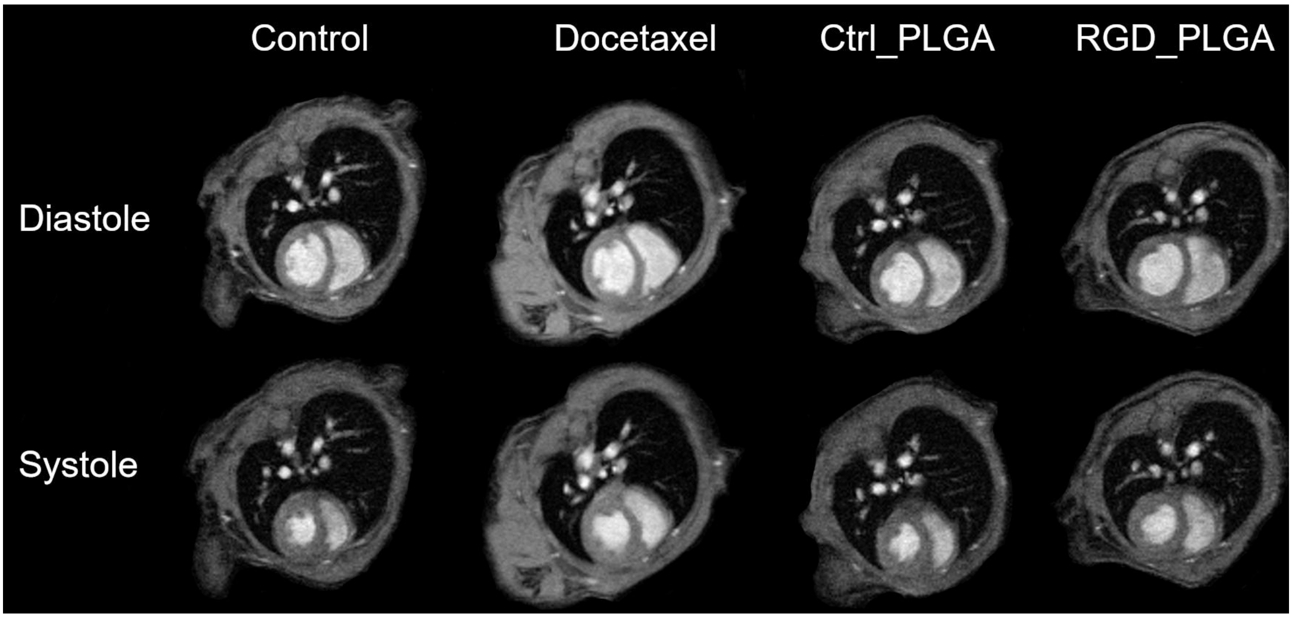

Figure S6. Representative diastolic or systolic axial MRI images of heart of BALB/c mice orthotopically challenged with $1x10^4$ 4T1 cells and treated with physiological saline solution as a control, free Docetaxel, Ctrl_PLGA or RGD_PLGA, C (N = 6 per group) (Flash cine cardioMR images).
